# Computational exploration of the reaction mechanism of the Cu^+^-catalysed synthesis of indoles from *N*-aryl enaminones

**DOI:** 10.1098/rsos.150582

**Published:** 2016-02-03

**Authors:** Carlos E. P. Bernardo, Pedro J. Silva

**Affiliations:** FP/ENAS, Faculdade de Ciências da Saúde, Universidade Fernando Pessoa, Rua Carlos da Maia, 296, Porto 4200-150, Portugal

**Keywords:** C–C coupling, Cu(I) reactivity, C–H bond activation

## Abstract

We have studied the role of Cu^+^-phenantroline as a catalyst in the cyclization of *N*-aryl-enaminones using density-functional theory computations. The catalyst was found to bind the substrate upon deprotonation of its eneaminone, and to dramatically increase the acidity of the carbon adjacent to the ketone functionality. The deprotonation of this carbon atom yields a carbanion which attacks the aryl moiety, thereby closing the heterocycle in the rate-determining step. This C–C bond forming reaction was found to proceed much more rapidly when preceded by re-protonation of the substrate N-atom (which had lost H^+^ in the initial step). Hydride transfer to the catalyst then completes the indole synthesis, in a very fast step. The influence of Li^+^ and K^+^ on the regio-selectivity of the cyclization of bromo-substituted analogues could not, however, be reproduced by our model. Alternative pathways involving either single-electron transfer from the catalyst to the substrate or ring cyclization without previous carbon *α*-deprotonation were found to be kinetically or thermodynamically inaccessible.

## 1. Introduction

**Scheme 1.**
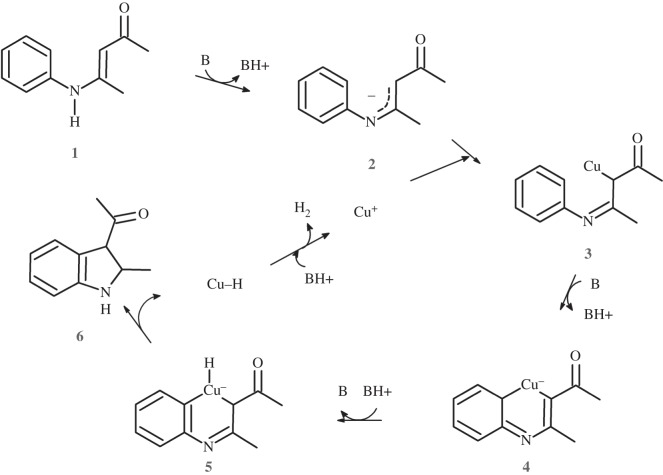
Reaction mechanism proposed by Bernini *et al.* [3] The phenanthroline ligand has been omitted for clarity.

Indoles are present in a wide variety of natural and synthetic products with extensive and diverse biological effects. Although many experimental protocols describing the synthesis of indole derivatives are available [1,2], considerable interest in the search for new routes to this molecular scaffold still remains, since many common reaction routes involve the use of expensive or toxic palladium catalysts, or require starting reagents of limited availability, such as *ortho*-substituted anilines. In 2009, Bernini *et al.* described a very attractive synthesis of indoles using *N*-aryl-eneaminones as starting material and inexpensive phenanthroline-bound Cu^+^ as a catalyst [3]. The Bernini synthesis is tolerant of a wide variety of substituents in the aryl ring and in the enaminone moiety, which enables the efficient production of highly substituted indoles. The reaction mechanism proposed in the original publication (scheme 1) proceeds through the sequential deprotonation of the eneaminone (**1**) and complexation of the resulting carbanion (**2**) by Cu^+^. Removal of an additional proton from the Cu-bound carbon position was then proposed to yield a transient tetra-coordinated Cu (**4**) bound to the enaminone, a phenanthroline ligand and the substrate aryl substituent. Electronic rearrangement would then enable the Cu ion to acquire significant negative charge and to acquire a proton, finally yielding a cyclized indole and a copper hydride. Regeneration of the catalyst through H_2_ evolution completes the proposed cycle.

We have subjected this reaction proposal to extensive density-functional computations. The proposed tetra-coordinated geometry of **4** was found to be absent from the catalytic cycle. Instead, **4** contains a tricoordinated Cu^+^ ion and a lone electron pair on the Cu-coordinating carbon atom, which may form a bond to the aryl moiety either immediately or upon re-protonation of the nitrogen atom.

## 2. Computational methods

The reaction mechanism was investigated using four different functionals: the popular B3LYP [4–6] and the three functionals (PBEPW91 [7,8], PBE0 [9] and PBE1PW91 [7–9]) shown to afford the best geometric and/or energetic agreement with high-level CCSD(T) benchmarking computations in similar Cu^+^-containing model systems [10]. Geometry optimizations were performed with the Firefly [11] quantum chemistry package, which is partially based on the GAMESS (US) [12] code, using autogenerated delocalized coordinates [13]. The SBKJ pseudo-potential [14] (and associated basis set) was used for Cu, and a medium-sized basis set, 6-31G(d), for all other elements. IRC computations confirmed that the obtained transition states did connect the relevant reactant and product states. Zero-point and thermal effects on the free energies at 298.15 and 373.15 K were computed at the optimized geometries. Accurate DFT energies of the optimized geometries obtained with each density functional were then computed using the same functional using 6-311G(2d,p) for all elements except Cu, which used the s6-31G* basis set developed by Swart *et al.* [15]. All energy values described in the text include solvation effects in nitromethane (*ε*=35.9), computed using the polarizable continuum model [16–18] implemented in Firefly, as well as dispersion and repulsion interactions with the continuum solvent, which were computed using the method developed by Amovili & Mennucci [19]. For comparison, solvation effects were also computed in tetrahydrofuran (*ε*=7.6). Intra- and inter-molecular dispersion effects were computed with the DFT-D3 formalism developed by Grimme *et al.* [20]. As the *s*_*r*,6_ and *s*_8_ parameters for functionals PBEPW91 and PBE1PW91 were not computed in the original publication, we determined them using the S22 reference data [21] present in the Benchmark Energy and Geometry Database [22]. *s*_*r*,6_ equals 1.1 for both functionals, whereas *s*_8_ varies from 0.37 (PBE1PW91) to 0.51 (PBEPW91). DFT-D3 corrections for these functionals are remarkably insensitive to the precise values of these parameters in the range 1.07<*s*_*r*,6_<1.15 and 0.3<*s*_8_<0.6.

## 3. Results and discussion

**Figure 1.**
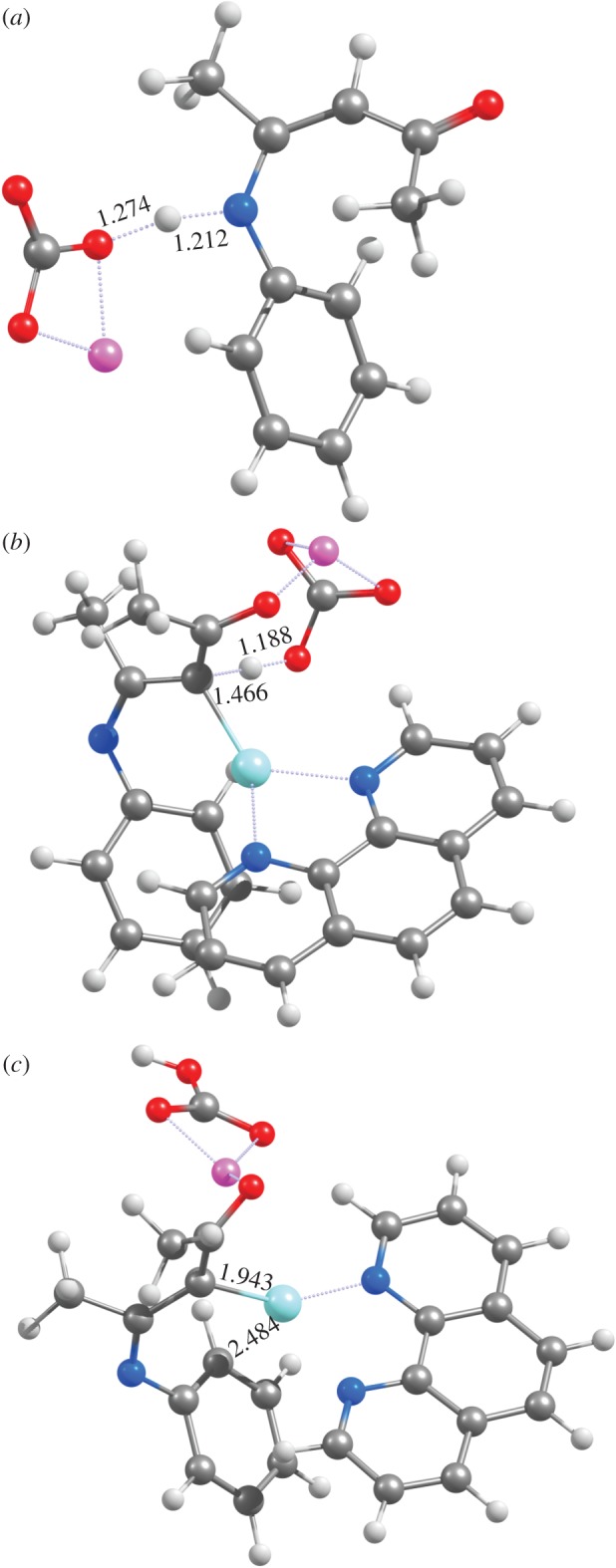
Optimized geometries of (*a*) **TS**_1_, (*b*) **TS**_2_ and (*c*) **4** at the PBE1PW91/6-31G(d) + SBKJ level of theory.

The proposed reaction mechanism relies on the use of a base to abstract two protons from the *N*-aryl eneaminone substrate. Experimentally, lithium carbonate was found to provide better reaction yields than alternative bases [3], and was therefore chosen as the general base in our computations. We used the ${\text{LiCO}}_{3}^{-}$ species instead of the neutral Li_2_CO_3_ form, as the high dielectric constant of the experimental solvent (dimethylformamide, *ε*=36.7) strongly favours the dissociation of ionic species. Our computations with all density functionals tested showed that the initial deprotonation of the *N*-aryl-enaminone **1** by lithium carbonate proceeds though a low-lying transition state (figure 1*a*) and therefore quickly reaches equilibrium under experimental conditions (table 1). As expected, removal of the C2 proton instead of the nitrogen proton is thermodynamically disfavoured, and would require surmounting a respectable kinetic barrier (21–22.5 kcal mol^−1^). After deprotonation of the nitrogen atom, direct removal of the carbonyl C_*α*_-hydrogen in **2** by ${\text{LiCO}}_{3}^{-}$ is also thermodynamically unfavourable by 30–35 kcal mol^−1^ (electronic supplementary material, table S2), and would yield an enaminone strongly bound to the LiHCO_3_ product through its negatively charged oxygen and C2 atoms. By contrast, addition of Cu^+^(phen) to the deprotonated species **2** is very spontaneous and occurs readily without any barrier. The spontaneity of this reaction step is more sensitive to the choice of functional than the initial deprotonation: PBEPW91 predicts it to be 10 kcal mol^−1^ more favourable than B3LYP, whereas PBE0 and PBE1PW91 afford values in the middle of the range spanned by B3LYP and PBEPW91.

**Table 1.** Relative energies (kcal mol^−1^) versus isolated reactants of the intermediates and transition states in the initial stages of the reaction mechanism. (All values include DFT-D3dispersion corrections, zero-point vibrational energy effects at 373.15 K, andsolvation effects in nitromethane.)

	B3LYP	PBE0	PBE1PW91	PBEPW91
**1**	1.3	−0.3	−0.4	−4.7
**TS**_1_	0.7	0.5	0.5	−4.1
**2**	0.3	−1.5	−1.3	−2.6
**3**	−25.1	−34.5	−23.8	−38.6
$\text{3}+{\text{LiCO}}_{3}^{-}$	−28.5	−35.2	−30.9	−38.9
**TS**_2_	−7.7	−13.3	−10.4	−19.7
**4**	−26.0	−31.6	−25.5	−37.4

The optimized geometries of intermediate **3** obtained with the different functionals show that these energetic trends are related to the interaction between the metal ion and the aromatic ring of the substrate: in PBE0 and PBE1PW91 the Cu^+^ ion lies 2.33 Å from the phenyl ring, whereas in PBEPW91 (the functional predicting the most favourable reaction) this distance has shortened to 2.22 Å and in B3LYP (the functional predicting the least spontaneous reaction), Cu^+^ sits quite distant (2.77 Å) from the ring. The deprotonation of the carbonyl C_*α*_-carbon atom of the substrate (figure 1*b*) by ${\text{LiCO}}_{3}^{-}$ is now possible with a relatively modest activation ΔG of 19–22 kcal mol^−1^ (depending on the functional), owing to the possibility of electronic charge redistribution onto the Cu^+^-containing ligand. This reaction step proceeds through a relatively ‘late’ transition state, where the Li^+^ ion is coordinated by the carbonate ion and by the substrate ketone moiety and, most importantly, the Cu^+^-carbon distance has decreased dramatically to 1.97–1.98 Å(irrespective of the chosen functional), while the leaving proton is only tenuously bound to the eneaminone (≈1.46 Å). The Cu^+^-carbon bond decreases only slightly to 1.91–1.96 Åas this reaction step proceeds to completion and the proton completely moves to the carbonate. The reaction product **4** (figure 1*c*) is predicted by B3LYP, PBE0 and PBE1PW91 to be bound to Cu(phen)^+^ through only one carbon atom, in contrast with Bernini’s proposal (scheme 1). The remaining functional (PBEPW91) predicts Cu(phen)^+^ to bind the doubly-deprotonated eneaminone through an additional interaction with the *ortho*-position of the aromatic ring, as advocated by Bernini.

**Scheme 2.**
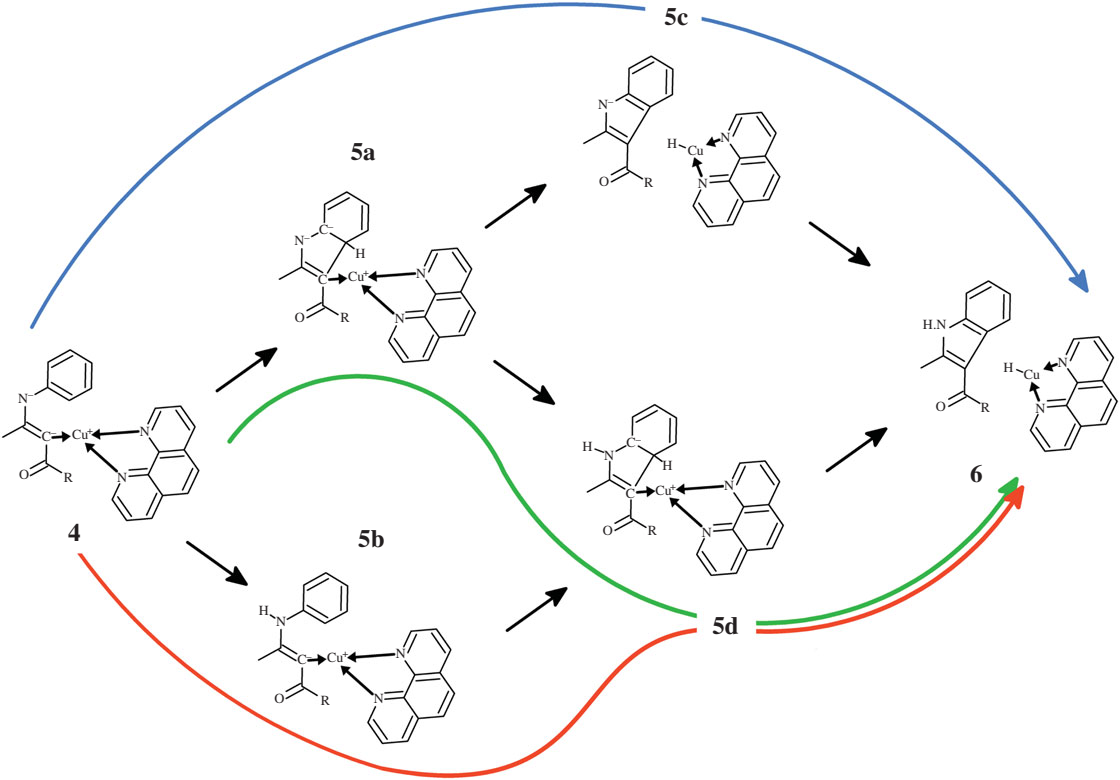
Reaction pathways leading from intermediate **4** to indole product.

Several distinct reaction steps must occur in the transformation of intermediate **4** into product indole **6**: proton capture by the eneaminone nitrogen, hydride ejection by the aryl substituent and closure of the five-membered pyrrole ring of the indole. As none of the putative intermediates has been observed experimentally, and the relative sequence of these reaction steps has therefore not been ascertained, we have analysed the different possible reaction sequences leading from **4** to **6** (scheme 2).

**Figure 2.**
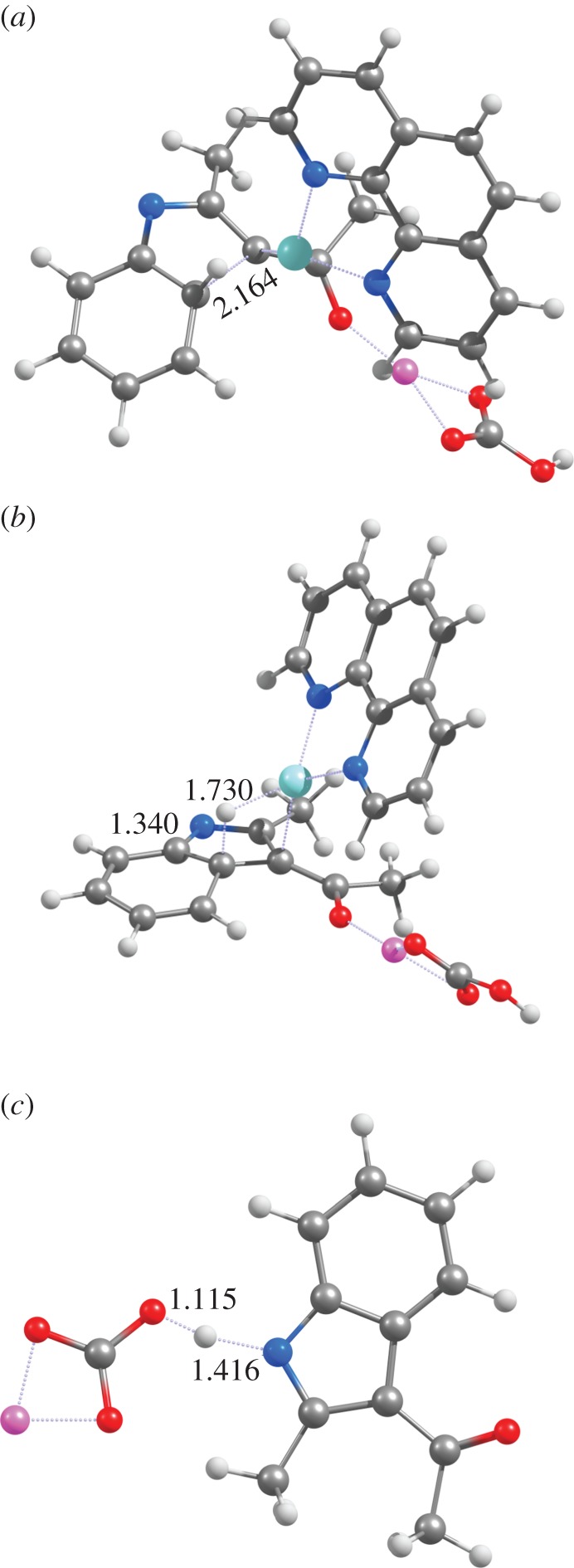
Optimized geometries of (*a*) **TS**_**4**→**5a**_, (*b*) **TS**_**5a**→**5c**_ and (*c*) **TS**_**5c**→**6**_ at the PBE1PW91/6-31G(d) + SBKJ level of theory (pathway depicted in blue in scheme 2).

Immediate ring closure of intermediate **4** (yielding intermediate **5a**) proceeds through a transition state with a very high activation free energy (46–56 kcal mol^−1^ for all functionals except PBEPW91, which has a ΔG^‡^=41.1 kcal mol^−1^). In this transition state (figure 2*a*), the distance between the carbons in the nascent C–C bond is very similar in all tested functionals (2.16 Åfor PBE0 and PBE1PW91; 2.19 Åfor PBEPW91 and B3LYP). Interestingly, the similarity between PBEPW91 and B3LYP does not extend to the reaction energetics: the activation free energy with PBEPW91 is lower than B3LYP by 14.6 kcal mol^−1^. Irrespective of functional, intermediate **5a** lies ≈32 kcal mol^−1^ below the preceding transition state.

**Figure 3.**
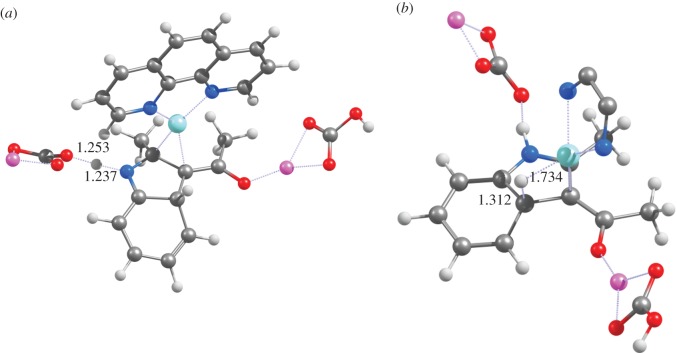
Optimized geometries of (*a*) **TS**_**5a**→**5d**_ and (*b*) **TS**_**5d**→**6**_ at the PBE1PW91/6-31G(d) + SBKJ level of theory (pathway depicted in green in scheme 2). In (*b*), the phenanthroline ligand has been partially deleted, for ease of viewing.

Subsequent hydride transfer from the hexagonal ring in **5a** to the Cu^+^-phenantroline catalyst (figure 2*b*) is a very fast and spontaneous process (activation ΔG<10 kcal mol^−1^; reaction ΔG ≈−11 to −26 kcal mol^−1^). Protonation of the resulting **5c** intermediate by LiHCO_3_ then occurs through a diffusion-controlled process with negligible (less than 2.5 kcal mol^−1^) activation energies. An alternative pathway arising from the **5a** intermediate is possible, by protonating the **5a** intermediate (figure 3*a*) before the hydride is transferred to the catalyst (figure 3*b*). This **5a** →**5d** →**6** sequence occurs, however, with a slightly higher activation free energy (≈13 kcal mol^−1^) than the **5a** →**5c** →**6** hydride transfer alternative. It is therefore likely that, if the reaction sequence arising from intermediate **4** proceeds through an initial ring closure (step **4** →**5a**), hydride transfer to the catalyst precedes the protonation of the nitrogen atom of the heterocycle. In both alternatives and using any of the tested functionals, the initial formation of the C–C bond between the aryl ring and the Cu^+^-bound eneaminone carbon (**4** →**5a**) is the rate-determining step.

**Figure 4.**
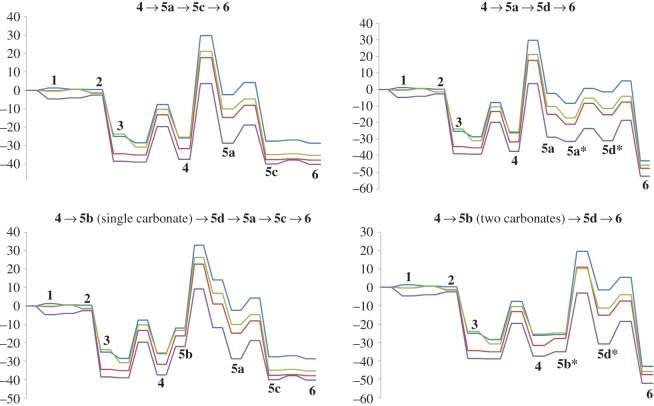
Potential energy surfaces of the different mechanisms studied in this work. Blue: B3LYP; green: PBE1PW91; red: PBE0; violet: PBEPW91. Species with an asterisk contain two molecules of lithium carbonate.

The high activation energy of the **4** →**5a** cyclization reaction step makes intermediate **4** prone to react through different pathways with lower activation free energies: specifically, protonation of the nitrogen atom in intermediate **4** may occur very easily upon addition of a second molecule of LiHCO_3_. Closure of the indole ring in the N-protonated state is more favourable (by 9–13 kcal mol^−1^) than observed in the unprotonated state. The nascent C–C bond in this transition state is much smaller than observed in the unprotonated state (1.95–1.96 Å versus 2.16–2.19 Å), and the total barrier (i.e. taking account of the energetic cost of bringing LiHCO_3_ from infinity to the proximity of **4**) remains below the barrier computed for the **4** →**5a** step (table 2 and figure 4). As in the previous reaction steps, PBEPW91 predicts the lowest activation-free energies and B3LYP the highest. PBE0 again affords very similar results to PBE1PW91. Indole synthesis is completed through hydride transfer from **5d** to the Cu^+^(phen) catalyst, which restores the aromatic system, as in the previously discussed **4** →**5a** →**5d** →**6** mechanism.

**Table 2.** Relative energies (kcal mol^−1^) versus isolated reactants of the intermediates and transition states in the evaluated reaction mechanisms (using a single carbonate). (All values include DFT-D3dispersion corrections, zero-point vibrational energy effects at 373.15 K, and solvation effects in nitromethane.)

1st step	2nd step	3rd step	B3LYP	PBE0	PBE1PW91	PBEPW91
**4**			−26.0	−31.6	−25.5	−37.4
TS **4** →**5a**			29.7	17.7	21.2	3.7
**5a**			−2.3	−14.9	−10.1	−28.8
	TS **5a** →**5c**		4.3	−8.1	−4.7	−18.8
	**5c**		−27.7	−37.6	−35.0	−40.1
		TS **5c** →**6**	−27.0	−37.2	−34.4	−38.0
		**6**	−28.7	−37.9	−35.3	−40.2
	**5a** + extra LiHCO_3_		−8.4	−20.9	−17.0	−31.3
	TS **5a**→**5d**+extra LiHCO_3_		0.7	−8.3	−5.1	−23.6
	**5d** + extra LiHCO_3_		−1.4	−15.1	−11.4	−30.8
TS **4**→**5b**+			no TS	no TS	no TS	no TS
extra LiHCO_3_						
**5b** + extra LiHCO_3_			−25.6	−27.8	−24.7	−35.0
	TS **5b**→**5d**+extra LiHCO_3_		19.4	11.0	10.0	−3.2
	**5d** + extra LiHCO_3_		−1.4	−15.1	−11.4	−30.8
		TS **5d**→**6**+	5.3	−7.5	−4.1	−18.6
		extra LiHCO_3_				
		**6** + extra LiHCO_3_	−43.0	−47.5	−45.7	−52.3

**Figure 5.**
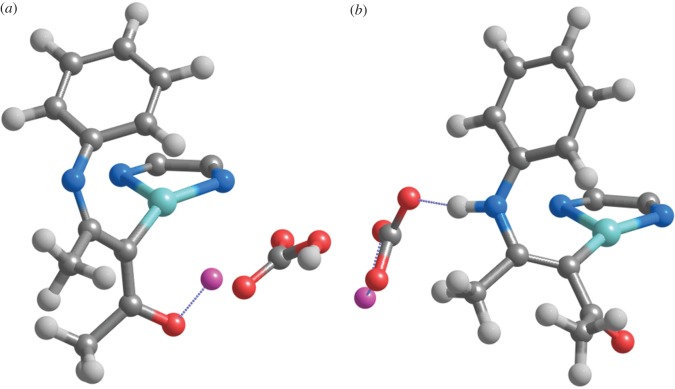
Optimized geometries of (*a*) **4**+LiHCO_3_ and (*b*) $\text{5b}+{\text{LiCO}}_{3}^{-}$ at the PBE1PW91/6-31G(d) + SBKJ level of theory (pathway depicted in green in scheme 2). The phenanthroline ligand has been partially deleted, for ease of viewing.

In the absence of a second molecule of base, conversion of **4** into **5b** is less favoured, since the proton donation must be effected by the LiHCO_3_ molecule formed in the deprotonation of **3** →**4**. This lone LiHCO_3_ species is, however, strongly stabilized through ionic interactions between its Li^+^ cation and the ketone moiety of the eneaminone (figures 1*c* and 5*a*), so that geometrical rearrangement of LiHCO_3_ into a position where it may donate a proton to the eneaminone nitrogen (figure 5*b*) is energetically quite costly (11–14 kcal mol^−1^). This energetic cost more than offsets the energetic advantage provided by the protonation of the nitrogen atom in the ring-closing step, leading to an overall barrier slightly higher than that observed in the original **4** →**5a** →**5d** pathway. Moreover, the lack of a stabilizing cation in the ketone moiety now causes the nitrogen atom in the nascent **5d** intermediate to spontaneously lose its proton to the nearby ${\text{LiCO}}_{3}^{-},$ leading to the generation of the **5a** intermediate, instead of **5d**.

The analysis presented above clearly shows that the **4** →**5b** →**5d** →**6** pathway is favoured over the **4** →**5a** →**5d** →**6** pathway. The barriers predicted by the different functionals are, however, relatively high: indeed, even the smallest barrier (35.7 kcal mol^−1^, computed with PBEPW91) at 100°C is still 5–6 kcal mol^−1^ above the barrier expected for a reaction with a half-life of 24 h.

**Figure 6.**
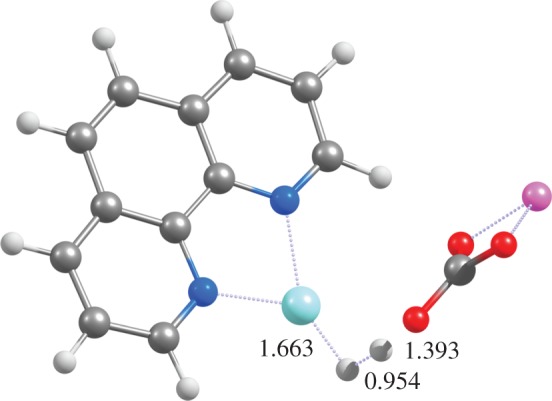
Optimized geometry of the transition state of H_2_ evolution from Cu^+^ (phen)-hydride at the PBE1PW91/6-31G(d) + SBKJ level of theory.

As the substrate is converted into an indole, Cu^+^(phen) is transformed into a Cu-hydride. Regeneration of the active Cu^+^(phen) is now needed to allow further rounds of catalysis. Bernini *et al.* suggested that this could be performed through simple H_2_ evolution from the Cu-hydride. Our computations do indeed show that this proposal is correct, as this reaction is exergonic and the transition state of the hydride transfer to a proton (provided by LiHCO_3_) (figure 6) lies only 7.5–9.5 kcal mol^−1^ above the reactants.

**Scheme 3.**
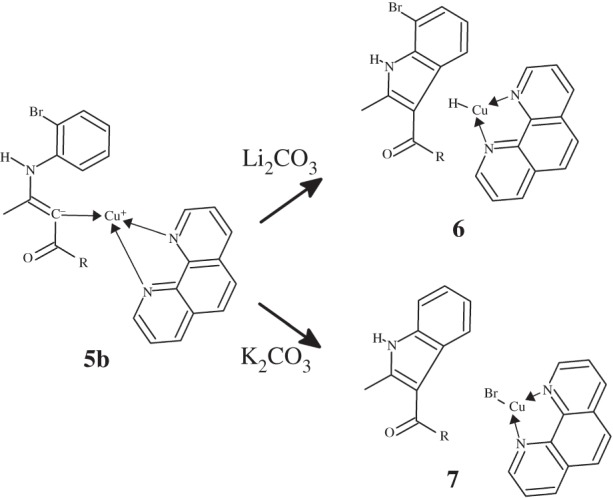
Cu^+^ -catalysed ciclization of *N*-2-bromophenyl-enaminone.

In the original experimental report, intriguing results were obtained when the reaction was performed on *N*-2-bromophenyl-enaminones: depending on the carbonate counter-cation, the carbon-based lone pair in **5b** could attack either the dehalogenated *o*-position of the ring, yielding a halogenated product **6**, or the bromo-containing phenyl carbon, which then led to the transfer of bromine to ${\text{Cu(phen)}}_{2}^{+}$ and the generation of the dehalogenated product **7** (scheme 3). Formation of **7** is strongly favoured in the presence of K^+^, whereas Li^+^ leads to the exclusive formation of **6**.

**Scheme 4.**
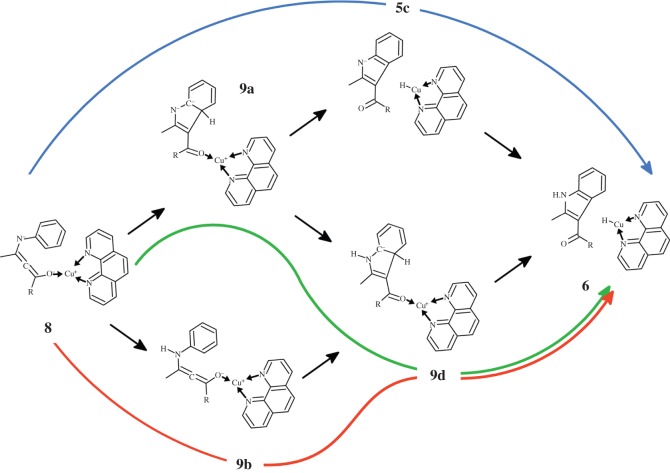
Reaction pathways leading from the intermediate with ketone-bound catalyst (**8**) to indole product.

We used the mechanistic pathways described above to study the conversion of Br-substituted substrates and found that the formation of the debrominated product **7** is strongly favoured by every functional, irrespective of the countercation used in the computation (table 3). Since the cation in these models binds the substrate ketone moiety and affects the reaction only through indirect effects on the electronic distribution across the aryl-enaminone conjugated system, we then hypothesized that catalyst migration from the carbon atom to the ketone moiety might provide a means of making the reaction more sensitive to the cation. Unfortunately, while this model (scheme 4) affords an easier attack of the aryl moiety by the carbon-based lone pair, it neither allows a faster overall mechanism (due to the higher energy of the ketone-bound species **8**, versus the original intermediate **4**) nor affords pathways where a Li^+^ countercation favours the retention of the halogen when a brominated substrate is used (electronic supplementary material, tables S3–S5).

**Table 3.** Relative energies (kcal mol^−1^) versus isolated reactants of the most characteristic transition states in the reaction of *o*-brominated substrates. (All values include DFT-D3 dispersion corrections, zero-point vibrational energy effects at 373.15 K, and solvation effects in nitromethane.)

	countercation(s)	B3LYP	PBE0	PBE1PW91	PBEPW91
TS **4** →**5a** (proximal Br)	Li^+^	22.0	34.2	20.5	15.7
TS **4** →**5a** (proximal Br)	K^+^	16.7	26.0	16.9	18.5
TS **4** →**5a** (distal Br)	Li^+^	53.9	51.5	51.8	41.4
TS **4** →**5a** (distal Br)	K^+^	49.2	57.3	42.2	38.1
TS **5b** →**7** (proximal Br)	Li^+^ Li^+^	21.8	27.9	22.7	23.7
TS **5b** →**7** (proximal Br)	Li^+^ K^+^	21.9	26.0	20.3	15.8
TS **5b** →**5d** (distal Br)	Li^+^ Li^+^	31.8	28.1	27.1	28.3
TS **5b** →**5d** (distal Br)	Li^+^ K^+^	35.9	38.2	31.6	34.7

The failure of our models in the description of the countercation effect on the reactivity of *o*-brominated substrate prompted a search for additional alternative pathways. Although it has been experimentally determined [3] that the reaction does not proceed when Cu^+^ is replaced by Cu^2+^, we wondered whether redox cycling of the Cu-based catalyst between the +1 and +2 oxidation states would allow the mechanism to circumvent one of the barriers found in the Cu^+^-catalysed mechanism through putative pathways occurring in the potential energy surface of the Cu^2+^-catalysed reaction, and eventually elicit susceptibility to the countercation through its influence in the redox cycling. All intermediates, however, proved to be remarkably resistant to reduction by Cu^+^-phenantroline (table 4), which rules out all possibilities of redox cycling.

**Table 4.** Free energies (kcal mol^−1^) of the reduction of key reaction intermediates by Cu^+^-phenantroline. (All values were obtained with the PBE0 functional, and include DFT-D3 dispersion corrections, zero-point vibrational energy effects at 373.15 K, and solvation effects in the given solvents.)

intermediate	in nitromethane (kcal mol^−1^)	in tetrahydrofuran (kcal mol^−1^)
**3**	72.5	92.8
**4**	91.3	121.4
**6**	72.2	95.5
**5a**	78.1	106.0
**5b**	63.3	88.3
**5d**	81.1	111.8

**Scheme 5.**
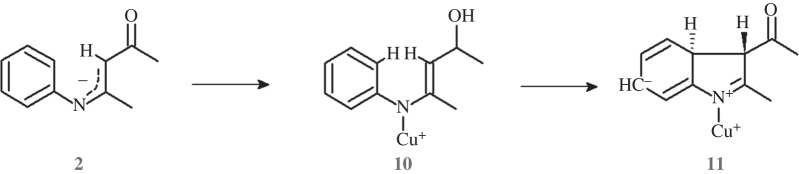
Putative mechanism leading to a reduced indole analogue through coordination of Cu^+^-phenatroline by deprotonated substrate, followed by immediate ring closure. The phenanthroline ligand has been omitted for clarity.

Following the suggestion of an anonymous reviewer, we also analysed whether the ring might close upon direct catalyst coordination to the deprotonated substrate, before either deprotonation of the carbon adjacent to the ketone functionality or hydride removal had occurred (scheme 5). This pathway is ruled out by the unfeasibly high activation-free energy of the **10** →**11** transition state (57 kcal mol^−1^, electronic supplementary material, table S6). Concerted transfer of the Cu^+^-phenantroline ligand from the N-atom to other positions in the heterocycle was also unable to afford lower-lying ring-closure transition states. These observations are not completely unexpected, as the experimentally observed inertness of the related *N*-aryl-enaminoates in the presence of Cu^+^-phenantroline [23] (which differ from the present substrates mostly by the lower acidity of the C_3_-bound hydrogens) strongly suggests that the deprotonation of the C_3_-carbon atom should either be rate-limiting itself, or else precede the rate-limiting ring-closing step.

## 4. Conclusion

**Scheme 6.**
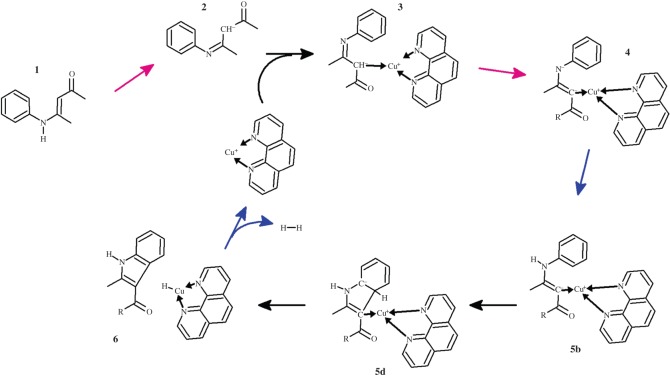
The most-favoured reaction mechanism for the Cu^+^-assisted indole synthesis. Rose arrows depict deprotonations by carbonate. Blue arrows depict reprotonations by hydrogencarbonate. The **5b** and **5d** states are most favoured when stabilized by two (hydrogen)carbonates, strongly suggesting that the best reaction rates will be observed when at least a twofold excess of base over N-aryl-enaminone is used.

The preceding results (scheme 6) highlight a dual role for the Cu^+^(phen) catalyst in this indole synthesis: on the one hand, its coordination to the deprotonated eneaminone is required to facilitate the deprotonation of the ketone *α*-carbon, yielding the carbanion that attacks the C_2_-position of the aryl moiety; on the other hand, Cu^+^ acts as the acceptor of the hydride ejected by the aryl C_2_-carbon in the re-aromatization step. In agreement with the experimentally observed absence of a deuterium isotope effect [3] the rate-determining step was found to correspond to the closure of the indole heterocycle through the formation of a C–C bond. Despite the relative success of the proposed mechanism, our computations cannot yet explain why *o*-brominated substrate fails to lose its halogen in the presence of a Li^+^ counteraction. Investigations of alternative pathways were also unfruitful. We hypothesize that cation–*π* interactions may play a role in the observed reaction outcome, as it is known that (at least in the gas phase) Li^+^ establishes stronger cation–*π* interactions with indoles and aryls than K^+^ [24–27] and such electronic effects may subtly influence the energetics of the *o*-brominated substrates. Unfortunately, solvent is known to dramatically affect the intensity of cation–*π* interactions [28], and therefore the computational exploration of this hypothesis is expected to become very time- and resource-consuming, as it requires the explicit inclusion of a sufficient number of solvent molecules around the cation (with the corresponding exponential increase of computational time). Experimental exploration of the effect of the cation on the reactivity of *o*-brominated substrates with varying degrees of polarization of the aryl system (using both electron-withdrawing and electron-donating substituents) (as exemplified in the research of Hunter *et al.* [29]) may, however, offer evidence regarding a possible role of cation–*π* interactions.

## Supplementary Material

molecules_in_tables_SI_tables_1_and_2_PBEPW91.xyz

## Supplementary Material

molecules_in_tables_SI_tables_1_and_2_PBE1PW91.xyz

## Supplementary Material

molecules_in_tables_SI_tables_1_and_2_PBE0.xyz

## Supplementary Material

molecules_in_tables_SI_tables_1_and_2_B3LYP.xyz

## Supplementary Material

molecules_in_SI_table_3_PBE0.xyz

## Supplementary Material

molecules_in_SI_table_3_B3LYP.xyz

## Supplementary Material

molecules_in_SI_table_3_PBE1PW91.xyz

## Supplementary Material

molecules_in_SI_table_3_PBEPW91.xyz

## Supplementary Material

molecules_in_SI_table_4_B3LYP.xyz

## Supplementary Material

molecules_in_SI_table_4_PBE0.xyz

## Supplementary Material

molecules_in_SI_table_4_PBE1PW91.xyz

## Supplementary Material

molecules_in_SI_table_4_PBEPW91.xyz

## Supplementary Material

molecules_in_SI_table_5_tab_4_to_8_to_9a_to_5c.xyz

## Supplementary Material

molecules_in_SI_table_5_tab_8_to_9a_to_5c_w_proximal_Br.xyz

## Supplementary Material

molecules_in_SI_table_5_tab_8_to_9b_to_9d_to_6_with_two_carbonates.xyz

## Supplementary Material

molecules_from_SI_table_5_tab_8_to_9a_w_distal_Br.xyz

## Supplementary Material

molecules_from_SI_table_5_tab 9b_to_9d_to_6_w_distal_Br_and_LiHCO3.xyz

## Supplementary Material

molecules_from_SI_table_5_tab_9b_to_9d_to_6_w_distal_Br_and_LiHCO3.xyz

## Supplementary Material

molecules_from_SI_table_5_tab_9b_to_9d_to_6_w_distal_Br_with_two_carbonates.xyz

## Supplementary Material

molecules_from_SI_table_5_tab_9b_to_9d_to_6_with_one_carbonate.xyz

## Supplementary Material

molecules_from_SI_table_5_tab_9b_to_9d_to_6_with_proximal_Br_and_carbonate.xyz

## Supplementary Material

molecules_from_SI_table_5_tab_9b_to_9d_to_6_with_proximal_Br_and_two_carbonates.xyz

## Supplementary Material

molecules_from_SI_table_5_with_KHCO3.xyz

## Supplementary Material

molecules in SI table 6.xyz

## Supplementary Material

SI_Table_1 reaction energies for main reaction, all pathways.xlsx

## Supplementary Material

SI_Table_2 Reaction energies for Cu-free conversion of 2 into 3.xls

## Supplementary Material

SI_table_3 Reaction energied for brominated 4 to 5a to 6 or 7.xls

## Supplementary Material

SI_table_4 Reaction Energies for brominated 5b to 6 or 7.xls

## Supplementary Material

SI_table_6_Reaction energies for scheme 5 and for reduction of key intermediates by Cu-phenantroline.xls

## Supplementary Material

SI_table_5 reaction Energies for brominated 4 to 8 to 9.xlsx

## Supplementary Material

SI_Table_7 reaction energies for Cu(phen) addition to 1.xlsx

## Data accessibility

Data for this paper can be found in the following repositories: http://dx.doi.org/10.6084/m9.figshare.1528305 and http://dx.doi.org/10.6084/m9.figshare.1528383.
